# Supplementary material for: A comparative mathematical modeling study of phenotypic approaches to T cell activation
Source: Sci Rep. 2025 Dec 20;15:44233. doi: 10.1038/s41598-025-32255-9 (PMC12722356; doi:10.1038/s41598-025-32255-9)
Supplement: Supplementary file 1 — Supplementary Information. [file 41598_2025_32255_MOESM1_ESM.pdf]

Supplementary Material

Yogesh Bali[1] and Alan D. Rendall*[1,2]

[1] Institut für Mathematik, Johannes Gutenberg-Universität, Staudingerweg 9, 55099 Mainz
[2]Institute for Quantitative and Computational Biosciences (IQCB), Johannes Gutenberg-Universität, Johannes-von-Müller-Weg 6, D-55128 Mainz

# 1 Nature of Solution

In this study, we also investigated the late-time behavior of our models, focusing on the existence and uniqueness of steady states and convergence properties as $t \to \infty$. Specifically, we address the question: Does each choice of parameters yield a unique steady state, and do all solutions converge to this steady state in the long term? Our findings are as follows:

1. For system (1) (Occupancy Model), the answer is affirmative. Given that this system is effectively one-dimensional, the proof of convergence to a unique steady state is straightforward.

2. For systems (2) and (6) (KPR and KPR with stabilizing activation chain), convergence to a unique steady state is also established, applying the theorem by Sontag, as referenced in prior communications.

3. For systems (3), (4), and (9) (KPR with limited signaling, sustained signaling, and both ), convergence to a unique steady state is confirmed based on the application of the Deficiency Zero Theorem, as demonstrated in the following sections.

4. For the system (5) (KPR with negative feedback ), however, the answer is negative, which aligns with the earlier findings.

5. Lastly, for systems (7) and (8) (KPR with IFF, and limited signalling), the answer is affirmative. The convergence to a unique steady state is proven below.

## 1.1 Kinetic Proofreading

Within this framework, a pMHC ligand (L) can bind reversibly to a TCR receptor (R), resulting in the formation of a pMHC-TCR complex denoted as $C_0$. Once formed, this TCR-pMHC complex ($C_0$) undergoes a series of biochemical changes to achieve a signaling-competent state represented as $C_N$. If a pMHC detaches from a TCR at any intermediate stage, all modifications are instantly undone, causing the TCR to return to its original, unmodified state. T cell activation is directly proportional to the quantity of TCRs in the $C_N$ state.

$$\frac{dL}{dt} = -\kappa LR - v\sum_{i=0}^{N} C_i$$

$$\frac{dR}{dt} = -\kappa LR - v\sum_{i=0}^{N} C_i$$

$$\frac{dC_0}{dt} = \kappa LR - (\phi + v)C_0$$

$$\frac{dC_i}{dt} = \phi C_{i-1} - (\phi + v)C_i; \quad 1 \leq i \leq N-1$$

$$\frac{dC_N}{dt} = \phi C_{N-1} - vC_N$$

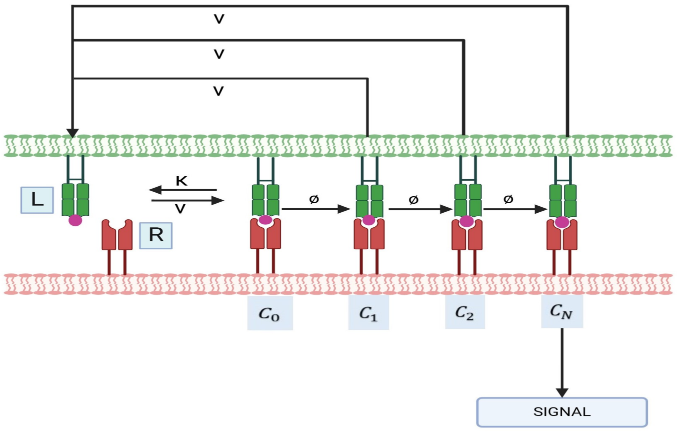


**Mathematical Formulation**
The above equations can be rewritten as:

$$\frac{dC_0}{dt} = \kappa(L_T - \sum_{i=0}^{N} C_i)(R_T - \sum_{i=0}^{N} C_i) - (\phi + v)C_0 \tag{1}$$

$$\frac{dC_i}{dt} = \phi C_{i-1} - (\phi + v)C_i; \quad 1 \leq i \leq N-1 \tag{2}$$

$$\frac{dC_N}{dt} = \phi C_{N-1} - vC_N \tag{3}$$

Here $R_T$ and $L_T$ are the total concentrations of the receptor and the ligand.
From equations (1)-(3), it can be deduced that if $\Sigma_1 = \sum_{i=0}^{N} C_i$

$$\frac{d\Sigma_1}{dt} = \kappa(L_T - \Sigma_1)(R_T - \Sigma_1) - v\Sigma_1 \tag{4}$$

**Lemma 1.1.** *Let $(C_0(t), C_1(t), \ldots, C_N(t))$ be a solution of (1)-(3) contained in the closure $K$ of the biologically relevant region. Then any $\omega$-limit point $(C_0^*, C_1^*, \ldots, C_N^*)$ of this solution is contained in the interior of $K$. In particular, any steady state is contained in the interior of $K$.*

*Proof.* For the proof we use Lemma 2.1(main text). It follows from equation (4) that $\sum_{i=0}^{N} C_i^*$ is strictly less than $L_T$ and $R_T$. It then follows from (1) that $C_0^* > 0$. This in turn implies using (2) and (3) that $C_i^* > 0$ for $1 \leq i \leq N$.

**Stability of the solutions**

In case of KPR, the binding reaction is of the form $L + R = C_0$. The bound receptor $C_0$ undergoes a series of phosphorylations to reach the signalling competent state $C_N$. Each $C_i$ can decay releasing $L$, $R$ and the phosphate groups. The network is weakly reversible. In our system, there are $n = N + 2$ complexes, and it contains only a single linkage class, which means $l = 1$. To show that the deficiency of the above weakly reversible network is zero it will be sufficient to show that the rank of the above system is $s = N + 1$.

There are $N + 2$ complexes $\{L + R, C_0, C_1, C_2, ...., C_N\}$.

The stoichiometric matrix is given by:

$$\begin{bmatrix} a_1 & a_2 & \ldots & a_{N+1} & b_1 & b_2 & \ldots & b_N & c_1 \\ 1 & 1 & \ldots & 1 & 0 & 0 & \ldots & 0 & -1 \\ 1 & 1 & \ldots & 1 & 0 & 0 & \ldots & 0 & -1 \\ -1 & 0 & \ldots & 0 & -1 & 0 & \ldots & 0 & 1 \\ 0 & -1 & \ldots & 0 & 1 & -1 & \ldots & 0 & 0 \\ \vdots & \vdots & \ddots & \vdots & \vdots & \vdots & \ddots & \vdots & \vdots \\ 0 & 0 & \ldots & -1 & 0 & 0 & \ldots & 1 & 0 \end{bmatrix}$$

The first $N+1$ columns of this matrix are linearly independent. Hence for this model $s \geq N+1$. Hence $\delta \leq (N+2) - (N+1) - 1 = 0$. Since $\delta$ is always non-negative this implies that $\delta = 0$.

## 1.2 Kinetic proofreading with limited signaling

This model extends the kinetic proofreading concept, suggesting that once a TCR reaches the signaling-competent state $C_N$, the bound TCR shifts to a non-signalling state $C_{N+1}$ at a rate $\xi$.

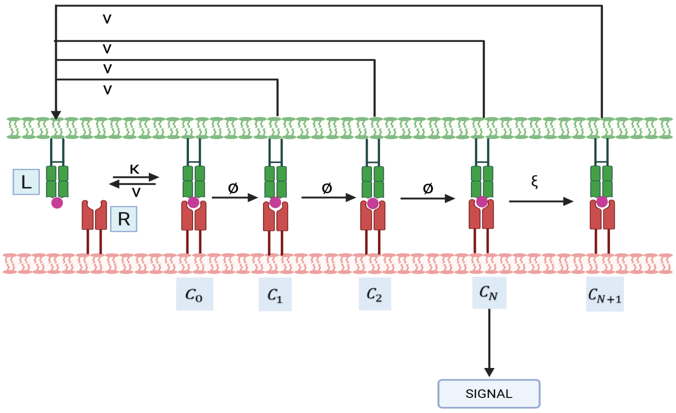


**Mathematical formulation**

$$\frac{dC_0}{dt} = \kappa(L_T - \sum_{i=0}^{N+1} C_i)(R_T - \sum_{i=0}^{N+1} C_i) - (\phi + v)C_0 \tag{5}$$

$$\frac{dC_i}{dt} = \phi C_{i-1} - (\phi + v)C_i; \quad 1 \leq i \leq N-1 \tag{6}$$

$$\frac{dC_N}{dt} = \phi C_{N-1} - (v + \xi)C_N \tag{7}$$

$$\frac{dC_{N+1}}{dt} = \xi C_N - vC_{N+1} \tag{8}$$

Here $R_T$ and $L_T$ are the total concentrations of receptor and the ligand.
Let $\Sigma_1 = \sum_{i=0}^{N+1} C_i$. Then, from Equations (5)-(8), we have:

$$\frac{d\Sigma_1}{dt} = \kappa(L_T - \Sigma_1^*)(R_T - \Sigma_1) - v\Sigma_1 \tag{9}$$

**Lemma 1.2.** *Let $(C_0(t), C_1(t), \dots, C_{N+1}(t))$ be a solution of (5)-(8) contained in the closure $K$ of the biologically relevant region. Then an $\omega$-limit point $(C_0^*, C_1^*, \dots, C_{N+1}^*)$ of this solution is contained in the interior of $K$. In particular, any steady state is contained in the interior of $K$.*

*Proof.* For the proof we use Lemma 2.1(main text). It follows from equation (9) that $\sum_{i=0}^{N+1} C_i^*$ is strictly less than $L_T$ and $R_T$. It then follows from (5) that $C_0^* > 0$. This in turn implies inductively, using (5)-(8) that $C_i^* > 0$ for $1 \leq i \leq N-1$.

We have proved the deficiency of this weakly reversible network is zero as in section **??**.

## 1.3 Kinetic proofreading with sustained signalling:

This model extends the KPR scheme by integrating experimental findings that indicate signalling-competent TCRs can maintain signaling for a defined time frame, even once pMHC unbinding occurs. In this framework, T-cell receptors (TCRs) in the signalling-capable state $C_N$ persist in signaling for a designated period ($T$) following the detachment of pMHC, subsequently returning to their baseline state at a rate $\lambda$.

**Mathematical Formulation**

$$\frac{dC_0}{dt} = \kappa(L_T - \sum_{i=0}^{N} C_i(t))(R_T - \sum_{i=0}^{N} C_i(t) - R^*(t)) - (v+\phi)C_0(t) \tag{10}$$

$$\frac{dC_i}{dt} = \phi C_{i-1}(t) - (\phi + v)C_i(t); \quad 1 \le i \le N-1 \tag{11}$$

$$\frac{dC_N}{dt} = \phi C_{N-1}(t) - vC_N(t) + \kappa(L_T - \sum_{i=0}^{N} C_i(t))R^*(t) \tag{12}$$

$$\frac{dR^*}{dt} = vC_N(t) - \kappa(L_T - \sum_{i=0}^{N} C_i(t))R^*(t) - \Omega R^*(t) \tag{13}$$

Here $R_T$ and $L_T$ are the total concentrations of receptors and the ligand.
If $\Sigma_1 = \sum_{i=0}^{N+1} C_i$ then it follows from equations (10)-(13) that:

$$\sum_{i=0}^{N} \frac{dC_i}{dt} = \kappa(L_T - \sum_{i=0}^{N} C_i)(R_T - \sum_{i=0}^{N} C_i - R^*) - v\sum_{i=0}^{N} C_i + \kappa(L_T - \sum_{i=0}^{N} C_i)R^* \tag{14}$$

and

$$\frac{d\Sigma_1}{dt} = \kappa(L_T - \Sigma_1)(R_T - \Sigma_1 - R^*) + \kappa R^*(L_T - \Sigma_1) - v\Sigma_1 \tag{15}$$

**Lemma 1.3.** *Let $(R^*(t), C_0(t), C_1(t), \ldots, C_N(t))$ be a solution of (10)-(13) contained in the closure $K$ of the biologically relevant region. Then any $\omega$-limit point $(R^{**}, C_0^*, C_1^*, \ldots, C_N^*)$ of this solution is contained in the interior of $K$. In particular, any steady state is contained in the interior of $K$.*

*Proof.* For the proof we use Lemma 2.1(main text). It follows from (15) that $\sum_{i=0}^{N} C_i^*$ is strictly less than $L_T$. Suppose now that $\sum_0^N C_i + R^{**} = R_T$. Then it follows from (10) that $C_0^* = 0$. This implies, using (11) that $C_i^* = 0$ for all $1 \le i \le N-1$. The sum of (12) and (13) then implies that $R^{**} = 0$. Substituting this back in (12) shows that $C_N^* = 0$. Putting these facts together we see that $\sum_{i=0}^{N} C_i + R^{**} = 0$, contradicting our assumption. Thus in fact $\Sigma_1 + R^{**} < R_T$. Once this has been established it follows from (10) that $C_0^* > 0$. Then (11) implies that $C_i^* > 0$ for $1 \le i \le N-1$. From (12) we can conclude that $C_N^* > 0$ and from (13) that $R^{**} > 0$.

**Stability of the Solutions of system:**

In the case of kinetic proofreading (KPR) with sustained signaling, the binding reaction follows $L + R = C_0$, where the bound receptor complex $C_0$ undergoes a sequence of phosphorylation steps to reach the signaling-competent state $C_N$. Unlike in limited signaling, here the TCRs continue to signal even after pMHC dissociates. The rate at which these unbound yet signaling-competent TCRs revert to their unmodified state is governed by a factor $\Omega$. In this model, T cell activation depends on both $C_N$ and $R^*$. Each intermediate state $C_i$ can decay, releasing $L$, $R$, $R^*$, and phosphate groups, making the network weakly reversible.

Our system consists of $n = N + 3$ complexes with a single linkage class ($l = 1$). To establish that the deficiency of this weakly reversible network is zero, it suffices to show that its rank is $s = N + 2$.

The network comprises $N + 3$ complexes: $\{L + R, C_0, C_1, C_2, \ldots, C_N, L + R^*\}$.

$$\begin{bmatrix} a_1 & a_2 & \dots & a_N & a_{N+1} & b_1 & b_2 & \dots & b_N & c_1 & c_2 & c_3 \\ 1 & 1 & \dots & 1 & 1 & 0 & 0 & \dots & 0 & -1 & -1 & 0 \\ 1 & 1 & \dots & 1 & 0 & 0 & 0 & \dots & 0 & -1 & 0 & 1 \\ -1 & 0 & \dots & 0 & 0 & -1 & 0 & \dots & 0 & 1 & 0 & 0 \\ 0 & -1 & \dots & 0 & 0 & 1 & -1 & \dots & 0 & 0 & 0 & 0 \\ \vdots & \vdots & \ddots & \vdots & \vdots & \vdots & \vdots & \ddots & \vdots & \vdots & \vdots & \vdots \\ 0 & 0 & \dots & -1 & 0 & 0 & 0 & \dots & -1 & 0 & 0 & 0 \\ 0 & 0 & \dots & 0 & -1 & 0 & 0 & \dots & 1 & 0 & 1 & 0 \\ 0 & 0 & \dots & 0 & 1 & 0 & 0 & \dots & 0 & 0 & -1 & -1 \end{bmatrix}$$

The first $N + 2$ columns of this matrix are linearly independent. Hence for this model $s \geq N + 2$. Hence $\delta \leq (N + 3) - (N + 2) - 1 = 0$. Since $\delta$ is always non-negative this implies that $\delta = 0$.

## 1.4 Kinetic Proofreading with Stabilizing Activation Chain

The model indicates that KPR complexes enhance the stability of foreign peptides while reducing their affinity for self-peptides. This selective strengthening and weakening of the $C_i$ complexes, as well as differences in activation timing, are represented through changes in the respective rate constants $v(i); (i = 0, 1, ..., N)$ and $\phi(i); (i = 0, 1, ..., N - 1)$ as the proofreading process advances.

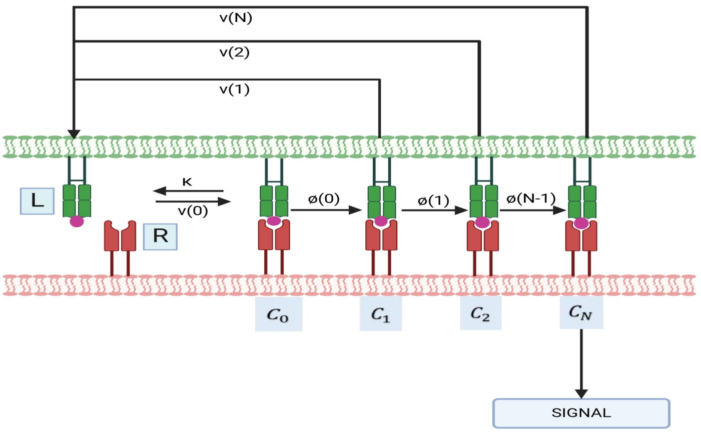


**Mathematical formulation**

$$\frac{dC_0}{dt} = \kappa(L_T - \sum_{i=0}^{N} C_i)(R_T - \sum_{i=0}^{N} C_i) - (\phi(0) + v(0))C_0 \tag{16}$$

$$\frac{dC_i}{dt} = \phi(i-1)C_{i-1} - (\phi(i) + v(i))C_i; \quad 1 \leq i \leq N - 1 \tag{17}$$

$$\frac{dC_N}{dt} = \phi(N-1)C_{N-1} - v(N)C_N \tag{18}$$

In this model for simplicity $v(0)$ is denoted as $v(= 1/\tau)$ which is dissociation time, and $\phi(0)$ denotes the propagation rate for the first step $C_0 \to C_1$. Now for next steps these rates are given by

$$v(i) = \frac{(1+i)}{(1+ri)}v; r > 1 \qquad \phi(i) = \phi r^i; r > 1$$

Let $\Sigma_1 = \sum_{i=0}^{N} C_i$. From Equations (16)-(18), we get:

$$\frac{d\Sigma_1}{dt} = \kappa\left(L_T - \Sigma_1\right)\left(R_T - \Sigma_1\right) - \Sigma_1 \sum_{i=0}^{N} v(i) \tag{19}$$

**Lemma 1.4.** *Let* $(C_0(t), C_1(t), \ldots, C_N(t))$ *be a solution of (16)-(18) contained in the closure* $K$ *of the biologically relevant region. Then any* $\omega$*-limit point* $(C_0^*, C_1^*, \ldots, C_N^*)$ *of this solution is contained in the interior of* $K$*. In particular, any steady state is contained in the interior of* $K$*.*

*Proof.* For the proof we use Lemma 2.1(main text). It follows from (19) that $\sum_{i=0}^{N} C_i^*$ is strictly less than $L_T$ and $R_T$. It then follows from (16) that $C_0^* > 0$. This in turn implies using (17) and (18) that $C_i^* > 0$ for $1 \leq i \leq N$.

**Stability of the solutions**

In the case of KPR with a stabilizing activation chain, the binding reaction follows $L+R = C_0$, where the bound receptor complex $C_0$ undergoes a sequence of phosphorylation steps to reach the signaling-competent state $C_N$. T cell activation is determined by $C_N$. Each intermediate state $C_i$ can decay, leading to the release of $L$, $R$, and phosphate groups, making the network weakly reversible.

Our system consists of $n = N + 2$ complexes with a single linkage class ($l = 1$). To establish that the deficiency of this weakly reversible network is zero, it suffices to show that its rank is $s = N + 1$.

The network comprises $N + 2$ complexes: $\{L + R, C_0, C_1, C_2, \ldots, C_N\}$.

The stoichiometric matrix is given by:

$$\begin{bmatrix} a_1 & a_2 & \ldots & a_{N+1} & b_1 & b_2 & \ldots & b_N & c_1 \\ 1 & 1 & \ldots & 1 & 0 & 0 & \ldots & 0 & -1 \\ 1 & 1 & \ldots & 1 & 0 & 0 & \ldots & 0 & -1 \\ -1 & 0 & \ldots & 0 & -1 & 0 & \ldots & 0 & 1 \\ 0 & -1 & \ldots & 0 & 1 & -1 & \ldots & 0 & 0 \\ \vdots & \vdots & \ddots & \vdots & \vdots & \vdots & \ddots & \vdots & \vdots \\ 0 & 0 & \ldots & -1 & 0 & 0 & \ldots & 1 & 0 \end{bmatrix}$$

The first $N + 1$ columns of this matrix are linearly independent. Hence for this model $s \geq N + 1$. Hence $\delta \leq (N + 2) - (N + 1) - 1 = 0$. Since $\delta$ is always non-negative this implies that $\delta = 0$.

## 1.5 Kinetic Proofreading with Limited and Sustained Signaling

This model is a combination of two models KPR with limited and KPR with sustained signaling.

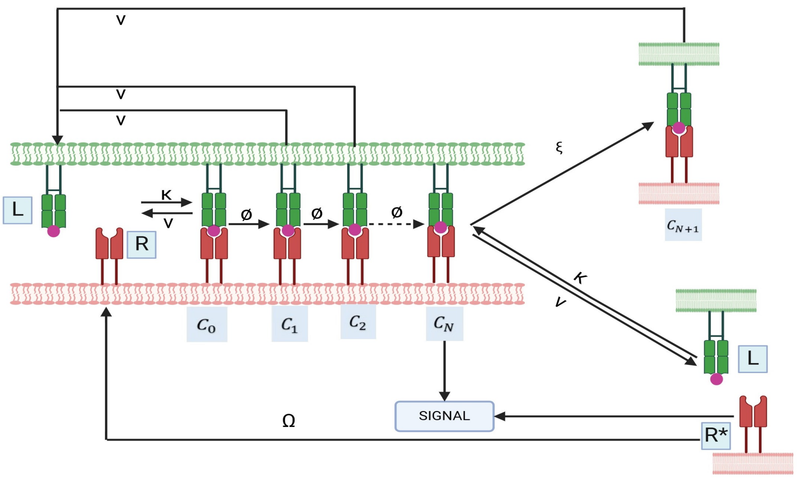


**Mathematical formulation**

$$\frac{dC_0}{dt} = \kappa(L_T - \sum_{i=0}^{N+1} C_i)(R_T - \sum_{i=0}^{N+1} C_i - R^*) - (\phi + v)C_0 \tag{20}$$

$$\frac{dC_i}{dt} = \phi C_{i-1} - (\phi + v)C_i; \quad 1 \leq i \leq N - 1 \tag{21}$$

$$\frac{dC_N}{dt} = \phi C_{N-1} - (v+\xi)C_N + \kappa(L_T - \sum_{i=0}^{N+1} C_i)R^* \quad (22)$$

$$\frac{dC_{N+1}}{dt} = \xi C_N - vC_{N+1} \quad (23)$$

$$\frac{dR^*}{dt} = vC_N - \kappa(L_T - \sum_{i=0}^{N+1} C_i)R^* - \Omega R^* \quad (24)$$

Define $\Sigma_1 = \sum_{i=0}^{N+1} C_i$. From equations ( 20)-(23) it follows that:

$$\frac{d\Sigma_1}{dt} = \kappa\left(L_T - \Sigma_1\right)\left(R_T - \Sigma_1 - R^*\right) - v\sum_{i=0}^{N+1} C_i - \Omega R^* \quad (25)$$

**Lemma 1.5.** *Let $(C_0(t), C_1(t), \ldots, C_{N+1}(t), R^*(t))$ be a solution of (20)-(23) contained in the closure $K$ of the biologically relevant region. Then any $\omega$-limit point $(C_0^*, C_1^*, \ldots, C_{N+1}^*, R^{**})$ of this solution is contained in the interior of $K$. In particular, any steady state is contained in the interior of $K$.*

*Proof.* For the proof we use Lemma 2.1(main text). It follows from (25) that $\sum_{i=0}^{N+1} C_i$ is strictly less than $L_T$. Suppose now that $\sum_{i=0}^{N} C_i + R^{**} = R_T$. Then it follows from (20) that $C_0^* = 0$. This implies, using (21) that $C_i^* = 0$ for all $1 \le i \le N-1$. The sum of (22), (23) and (24) implies that $R^{**} = 0$. Substituting this back into (23) gives $C_{N+1} = 0$ and substituting this into (12) gives $C_N = 0$. Putting these facts together we see that $\sum_{i=0}^{N+1} C_i^* + R^{**} = 0$, contradicting our assumption. Thus in fact $\sum_{i=0}^{n+1} C_i + R^{**} < R_T$. Once this has been established it follows from (20) that $C_0^* > 0$. Then (21) implies that $C_i^* > 0$ for $1 \le i \le N-1$. From (22) we can conclude that $C_N^* > 0$, from (23) that $C_{N+1}^* > 0$ and from (24) that $R^{**} > 0$.

**Stability of the solutions**

In case of KPR with limited and sustained signalling the binding reaction is of the form $L + R = C_0$. The bound receptor $C_0$ undergoes a series of phosphorylations to reach signalling competent state $C_N$. In this model, T cell activation is determined by both $C_N$ and $R^*$. Each $C_i$ can decay releasing $L$ ,$R$, $R^*$, and the phosphate groups. The network is weakly reversible. In our system we have $n = N+4$ complexes; there is only one linkage class i.e., $l = 1$. To show that the deficiency of the above weakly reversible network is zero. It will be sufficient to show that the rank of the above system is $s = N+3$.

There are $N+4$ complexes $\{L+R, C_0, C_1, C_2, ...., C_{N+1}, L+R^*\}$.

The stoichiometric matrix is given by:

$$\begin{bmatrix} a_1 & a_2 & \ldots & a_{N+2} & b_1 & b_2 & \ldots & b_{N+1} & c_1 & c_2 & c_3 \\ 1 & 1 & \ldots & 1 & 0 & 0 & \ldots & 0 & -1 & -1 & 0 \\ 1 & 1 & \ldots & 1 & 0 & 0 & \ldots & 0 & -1 & -1 & 1 \\ 0 & 0 & \ldots & 0 & 0 & 0 & \ldots & 0 & 0 & 0 & -1 \\ -1 & 0 & \ldots & 0 & -1 & 0 & \ldots & 0 & 1 & 0 & 0 \\ 0 & -1 & \ldots & 0 & 1 & -1 & \ldots & 0 & 0 & 0 & 0 \\ \vdots & \vdots & \ddots & \vdots & \vdots & \vdots & \ddots & \vdots & \vdots & \vdots & \vdots \\ 0 & 0 & \ldots & 0 & 0 & 0 & \ldots & -1 & 0 & 1 & 0 \\ 0 & 0 & \ldots & -1 & 0 & 0 & \ldots & 1 & 0 & 0 & 0 \end{bmatrix}$$

The first $N+3$ columns of this matrix are linearly independent. Hence for this model $s \ge N+3$. Hence $\delta \le (N+4) - (N+3) - 1 = 0$. Since $\delta$ is always non-negative this implies that $\delta = 0$.

### 1.6 Kinetic proofreading with limited signaling and incoherent feed forward loop

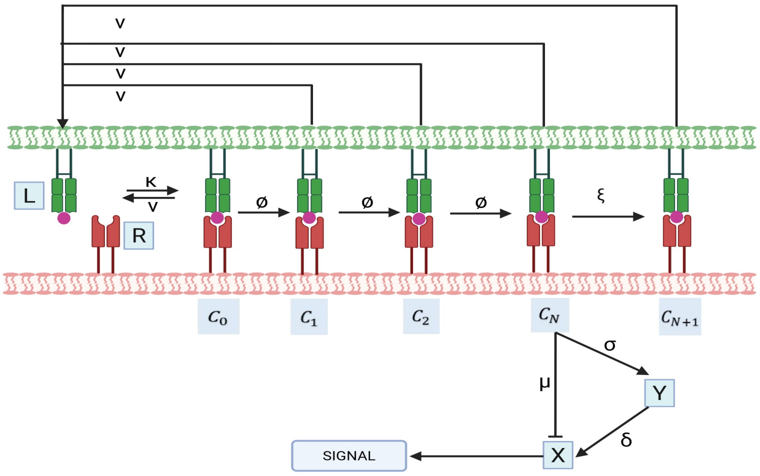


This model is an extension of KPR with an incoherent feed forward loop, it has been assumed that signalling is limited and after reaching the signalling competent state the bound TCR transits to a non-signalling transit state.

$$\frac{dC_0}{dt} = \kappa(L_T - \sum_{i=0}^{N+1} C_i)(R_T - \sum_{i=0}^{N+1} C_i) - (\phi + v)C_0() \tag{26}$$

$$\frac{dC_i}{dt} = \phi C_{i-1} - (\phi + v)C_i; \quad 1 \leq i \leq N-1 \tag{27}$$

$$\frac{dC_N}{dt} = \phi C_{N-1} - (v + \xi)C_N \tag{28}$$

$$\frac{dC_{N+1}}{dt} = \xi C_N - vC_{N+1} \tag{29}$$

$$\frac{dY}{dt} = a(m - Y) - bY + \sigma C_N(m - Y) \tag{30}$$

$$\frac{dX}{dt} = c(l - X) - dX + \delta Y(l - X) - \mu C_N X \tag{31}$$

We aim to demonstrate that the system, an extension of the kinetic proofreading (KPR) model, is globally asymptotically stable. The system consists of two sets of variables: $Z_1$, consisting of the $C_i$, which satisfies a closed system of equations

$$\frac{dZ_1}{dt} = f(Z_1),$$

and $Z_2$, consisting of $X$ and $Y$, governed by

$$\frac{dZ_2}{dt} = g(Z_1, Z_2).$$

It has already been proven that the system for $Z_1$ has a unique steady state, and that all solutions for $Z_1$ converge to this steady state as $t \to \infty$. In other words, as $t \to \infty$ each $C_i(t)$ converges to some $C_i^* > 0$. Consider any $\omega$-limit point of a solution $(C_i, X, Y)$ of (26)-(31) and the solution starting at that point for $t = 0$. It lies entirely in the $\omega$-limit set of that solution and so $C_i$ has the constant value $C_i^*$ for all $i$. This means that this solution satisfies the equations obtained from (30) and (31) by replacing $C_N$ by $C_N^*$. Equation (30) is an equation for $Y$ alone and so it it easy to see that the solution converges to $Y^* = \frac{a+b+\lambda C_N^*}{a+\lambda C_N^*}$ as $t \to \infty$. Now we can again pass to a solution starting at an $\omega$-limit point to see that

for the resulting solution $X$ solves the equation obtained from (31) by replacing $C_N$ by $C_N^*$ and $Y$ by $Y^*$. Thus the solution converges to $X^* = \frac{(c+\delta l)Y^*}{c+d+\delta Y^*+\mu C_N^*}$ as $t \to \infty$. Since these statements hold for all $\omega$-limit points it follows that any solution of (26)-(31) converges to $(C_i^*, Y^*, X^*)$ as $t \to \infty$. For this system the unique positive steady state is globally asymptotically stable.

## 1.7 Kinetic proofreading with incoherent feed forward loop

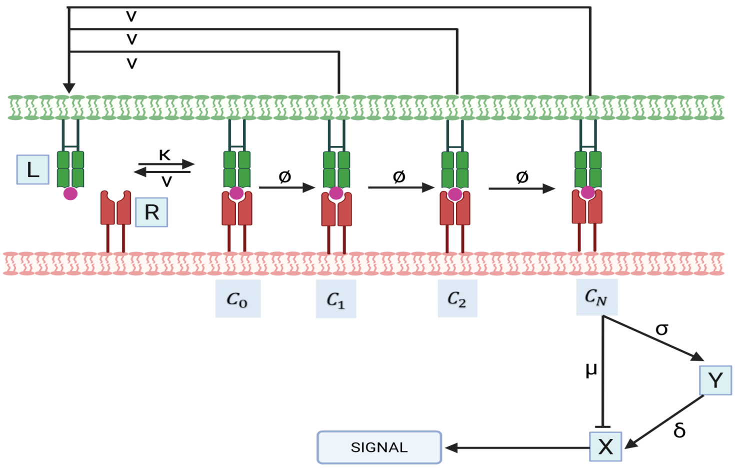


For this model there is a unique steady state in each stoichiometric compatibility class which is globally asymptotically stable. The proof of this is based on the fact that we have already proved global asymptotic stability for the kinetic proofreading model and otherwise the proof is just as in the previous example.

## 1.8 Analytical observations on results:

**Occupancy model :**

Response as a Monotonic Function of Dissociation Time:

In the context of the occupancy model, we analyze the response by examining T cell activation as a function of receptor-ligand complex concentration, $C$, which is derived from the equilibrium concentrations of ligand and receptor. It satisfies the equation:

$$C = \frac{L_T + R_T + K_D - \sqrt{(L_T + R_T + K_D)^2 - 4L_T R_T}}{2} \tag{32}$$

and is this model $C$ is a measure of the T cell activation.

$C$ is a solution to a quadratic equation of the form:

$$C^2 - aC + b = 0 \tag{33}$$

where

$$a = L_T + R_T + K_D > 0, \quad b = L_T R_T > 0$$

Only the negative square root gives a relevant solution, since $C < L_T$ and $C < R_T$ and therefore $C < a$.

The above equation can be reformulated as follows:

$$C = \frac{a - \sqrt{a^2 - 4b}}{2} \tag{34}$$

**Analysis of the solution**

Differentiating the expression for $C$ with respect to $K_D$ gives

$$\frac{dC}{dK_D} = \frac{\partial C}{\partial a} \cdot \frac{da}{dK_D}$$

$C$ is a decreasing function of $a$ since

$$\frac{\partial C}{\partial a} = \frac{1}{2}\left(1 - \frac{a}{\sqrt{a^2 - 4b}}\right) < 0$$

and combining this with

$$\frac{da}{dK_D} = 1 > 0$$

we have:

$$\frac{dC}{dK_D} < 0$$

**Relationship with Dissociation Time**

The dissociation constant $K_D$ is related to the dissociation rate constant $k_{\text{off}}$ and the association rate constant $k_{\text{on}}$:

$$K_D = \frac{k_{\text{off}}}{k_{\text{on}}}$$

Additionally, the dissociation time $\tau$ is inversely proportional to the dissociation rate constant:

$$\tau = \frac{1}{k_{\text{off}}}$$

As the dissociation time $\tau$ increases, the dissociation rate constant $k_{\text{off}}$ decreases, resulting in a decrease in $K_D$. Given that $C$ is a decreasing function of $K_D$, it follows that $C$ becomes an increasing function of the dissociation time $\tau$.

This relationship demonstrates that as ligand-receptor binding stabilizes, resulting in a longer dissociation time, T cell activation increases, highlighting the importance of bond longevity in cellular activation responses.

**Response as monotonic function of ligand concentration**

To determine how $C$ depends on $L_T$, we differentiate $C$ with respect to $L_T$:

$$\frac{dC}{dL_T} = \frac{\partial C}{\partial a} \cdot \frac{da}{dL_T} + \frac{\partial C}{\partial b} \cdot \frac{db}{dL_T}$$

We have $\frac{da}{dL_T} = 1$ and $\frac{db}{dL_T} = R_T$. Differentiating $C$ with respect to $b$ gives:

$$\frac{\partial C}{\partial b} = \frac{1}{2}\left(\frac{2}{\sqrt{a^2 - 4b}}\right)$$

Sign of $\frac{dC}{dL_T}$

Analyzing the derivative $\frac{dC}{dL_T}$:

$$\frac{dC}{dL_T} = \frac{1}{2}\left(1 - \frac{a}{\sqrt{a^2 - 4b}}\right) + \frac{R_T}{\sqrt{a^2 - 4b}} > 0.$$

Thus, the equilibrium concentration of the pMHC-TCR complex ($C$) is an increasing function of the ligand concentration ($L_T$). As the ligand concentration increases, the number of available binding sites increases, which directly enhances the formation of the complex ($C$). Therefore, in the occupancy model, the response function is positively dependent on the ligand concentration.

**Response of KPR model as increasing function of dissociation time**

In the kinetic proofreading model the response function is given by:

$$\text{Response} = a^N C_T$$

where

$$a = \frac{\phi}{(\phi + v)}$$

and

$$C_T = \frac{L_T + R_T + K_D - \sqrt{(L_T + R_T + K_D)^2 - 4L_T R_T}}{2}$$

Similar to the argument in occupancy model it is clear that for this model $C_T$ satisfies the same equation as $C$ does in the occupancy model. Thus it is a decreasing function of $k_{\text{off}}$, which follows that it is an is an increasing function of the dissociation time ($\tau$).

**Response of KPR model as increasing function of ligand concentration**

That the response with respect to ligand concentration is monotone in the kinetic proofreading model can be attributed again to the fact that in this case $C_T$ is increasing (similar to the argument in the case of the occupancy model ) and $\alpha$ is constant. Thus $R$ is increasing.

## 2 Mathematical Formulation and analysis

### 2.1 Occupancy Model

In this model a pMHC ligand (L) can reversibly bind a T cell receptor (R) to form a pMHC-TCR complex (C).

$$\frac{dC}{dt} = \kappa LR - vC \tag{35}$$

where

$$k_{\text{on}} = \kappa \qquad \text{and} \qquad k_{\text{off}} = v$$

and at equilibrium $\frac{dC}{dt} = 0$ and hence

$$LR = K_D C \tag{36}$$

where $K_D = \frac{v}{\kappa}$
Also the total amount of ligand $L_T$ and total amount of receptor TCR $R_T$ are conserved quantities

$$L_T = L + C \quad R_T = L + C \tag{37}$$

By inserting these in equation in (36) we get

$$C = \frac{L_T + R_T + K_D - \sqrt{(L_T + R_T + K_D)^2 - 4L_T R_T}}{2}$$

$$\text{T cell activation} = C$$

**Calculating the** $E_{\text{max}}$
The $E_{\text{max}}$ can then be found by finding $C$ in the limit of $L_T$ tending to infinity. In this limit, we have

$$\lim_{L_T \to \infty} \frac{K_D}{L_T} = 0 \quad \lim_{L_T \to \infty} \frac{R_T}{L_T} = 0$$

$$\begin{aligned}
E_{\text{max}} &= \lim_{L_T \to \infty} C \\
&= \lim_{L_T \to \infty} \frac{L_T + R_T + K_D - \sqrt{(L_T + R_T + K_D)^2 - 4L_T R_T}}{2} \\
&= \lim_{L_T \to \infty} \frac{2L_T R_T}{L_T + R_T + K_D + \sqrt{(L_T + R_T + K_D)^2 - 4L_T R_T}} \\
&= \lim_{L_T \to \infty} \frac{2R_T}{1 + \frac{R_T}{L_T} + \frac{K_D}{L_T} + \sqrt{(1 + \frac{R_T}{L_T} + \frac{K_D}{L_T})^2 - 4\frac{R_T}{L_T}}} \\
&= R_T
\end{aligned}$$

Hence

$$E_{\text{max}} = R_T$$

**Calculation for** $EC_{50}$
At half the maximal response, we have $L_T = EC_{50}$.
Also, T cell activation =

$$E_{\text{max}}/2 = R_T/2$$

which implies

$$C = R_T/2$$

Using equation (36) we have

$$(L_T - C)(R_T - C) = K_D C$$

hence

$$(L_T - R_T/2)(R_T - R_T/2) = K_D R_T/2$$

Rearranging we get

$$L_T = K_D + R_T/2$$

Hence

$$EC_{50} = K_D + R_T/2$$

## 2.2 Kinetic Proofreading

In this model, a pMHC ligand (L) can reversibly associate with a TCR receptor (R) to form a pMHC-TCR complex, denoted as $C_0$. Once formed, this complex undergoes a series of biochemical modifications, progressively transitioning towards a signaling-competent state, labeled as $C_N$. If the pMHC dissociates from the TCR at any intermediate stage, all modifications are rapidly undone, causing the TCR to revert to its original, unmodified state. The level of T cell activation is directly linked to the quantity of TCRs in the fully modified $C_N$ state.

$$\frac{dL}{dt} = -\kappa LR - v\sum_{i=0}^{N} C_i \tag{38}$$

$$\frac{dR}{dt} = -\kappa LR - v\sum_{i=0}^{N} C_i \tag{39}$$

$$\frac{dC_0}{dt} = \kappa LR - (\phi + v)C_0 \tag{40}$$

$$\frac{dC_i}{dt} = \phi C_{i-1} - (\phi + v)C_i; \quad 1 \le i \le N-1 \tag{41}$$

$$\frac{dC_N}{dt} = \phi C_{N-1} - vC_N \tag{42}$$

We have the total amount of ligand $L_T$ and total amount of receptor TCR $R_T$ as conserved quantities:

$$L_T = L + C_T \quad R_T = L + C_T$$

where $C_T = \sum_{i=0}^{N} C_i$

Using values of $L_T$ and $R_T$ in equation (38) we get

$$C_T = \frac{L_T + R_T + K_D - \sqrt{(L_T + R_T + K_D)^2 - 4L_T R_T}}{2} \quad \text{where} \quad K_D = \frac{v}{\kappa}$$

Let

$$a = \frac{\phi}{(\phi + v)}$$

Hence, at equilibrium

$$\begin{aligned}
C_T &= \sum_{i=0}^{N} C_i \\
&= \sum_{i=0}^{N-1} C_i + C_N \\
&= \sum_{i=0}^{N-1} a^i C_0 + C_N \\
&= \frac{(1-a^N)}{(1-a)} C_0 + C_N \\
&= \frac{(1-a^N)}{(1-a)} (1-a) C_T + C_N \\
&= \frac{1}{a^N} C_N
\end{aligned}$$

$$C_T = \frac{1}{a^N} C_N$$

Hence

$$\begin{aligned}
\text{T cell activation} &= C_N \\
&= a^N C_T
\end{aligned}$$

**Calculation for $E_{\max}$**

$$\begin{aligned}
E_{\max} &= \lim_{L_T \to \infty} C_T \\
&= \lim_{L_T \to \infty} \frac{L_T + R_T + K_D - \sqrt{(L_T + R_T + K_D)^2 - 4L_T R_T}}{2} \\
&= \lim_{L_T \to \infty} \frac{2L_T R_T}{L_T + R_T + K_D + \sqrt{(L_T + R_T + K_D)^2 - 4L_T R_T}} \\
&= \lim_{L_T \to \infty} \frac{2R_T}{1 + \frac{R_T}{L_T} + \frac{K_D}{L_T} + \sqrt{(1 + \frac{R_T}{L_T} + \frac{K_D}{L_T})^2 - 4\frac{R_T}{L_T}}} \\
&= R_T
\end{aligned}$$

So $E_{\max} = a^N R_T$

**Calculation for $EC_{50}$**

At half the maximal response, T cell activation =

$$E_{\max}/2 = a^N R_T/2$$

which implies

$$a^N C_T = a^N R_T/2$$

$$C_T = R_T/2$$

Since, $L_T = L + C_T \quad R_T = L + C_T$ Using this equation in equation (38)

$$\begin{aligned}\kappa(L_T - C_T)(R_T - C_T) - v\sum_{i=0}^{N} C_i = 0\\ (L_T - C_T)(R_T - C_T) = K_D C_T\\ L_T = K_D + R_T/2\end{aligned}$$

## 2.3 Kinetic proofreading with limited signaling

It is an extension of the kinetic proofreading model that proposes that when a TCR has reached signalling competent state $C_N$, the bound TCR transits to a non signalling state $C_{N+1}$ with rate $\xi$.

$$\frac{dL}{dt} = -\kappa LR - v\sum_{i=0}^{N+1} C_i \tag{43}$$

$$\frac{dR}{dt} = -\kappa LR - v\sum_{i=0}^{N+1} C_i \tag{44}$$

$$\frac{dC_0}{dt} = \kappa LR - (\phi + v)C_0 \tag{45}$$

$$\frac{dC_i}{dt} = \phi C_{i-1} - (\phi + v)C_i; \quad 1 \le i \le N-1 \tag{46}$$

$$\frac{dC_N}{dt} = \phi C_{N-1} - (v + \xi)C_N \tag{47}$$

$$\frac{dC_{N+1}}{dt} = \xi C_N - vC_{N+1} \tag{48}$$

Here $C_T = \sum_{i=0}^{N+1} C_i$, and T cell activation is given by $C_N$.

$$\begin{aligned}C_T &= \sum_{i=0}^{N-1} C_i + C_N + C_{N+1}\\ &= \sum_{i=0}^{N-1} a^i C_0 + C_N + \frac{\xi}{v} C_N\\ &= \frac{(1 - a^N)}{(1 - a)}(1 - a)C_T + \frac{v + \xi}{v} C_N\end{aligned}$$

Hence

$$C_T = \frac{1}{a^N}\left(\frac{v + \xi}{v}\right) C_N.$$

and

$$C_N = \left(\frac{v}{v+\xi}\right) a^N C_T$$

**Calculating the $E_{\max}$**
Similar to the case of kinetic proofreading model, it can be shown that when $L_T \to \infty$ $C_T \to R_T$
Hence

$$E_{\max} = \left(\frac{v}{v+\xi}\right) a^N R_T$$

**Calculating the $EC_{50}$**

At half the maximal response, T cell activation

$$\frac{E_{\max}}{2} = \left(\frac{v}{v+\xi}\right) a^N \frac{R_T}{2}$$

which implies

$$\left(\frac{v}{v+\xi}\right) a^N C_T = \left(\frac{v}{v+\xi}\right) a^N \frac{R_T}{2}$$

Hence

$$C_T = \frac{R_T}{2}$$

Since, $L_T = L + C_T \quad R_T = R + C_T$ Using this equation in (43)

$$\kappa(L_T - C_T)(R_T - C_T) - v\sum_{i=0}^{N+1} C_i = 0$$
$$(L_T - C_T)(R_T - C_T) = K_D C_T$$
$$L_T = K_D + R_T/2$$

## 2.4 Kinetic proofreading with sustained signaling

It is another modification of the kinetic proofreading model, according to this model, T-cell receptors (TCRs) in the signaling-competent state $C_N$, persist in signaling for a certain duration (T) after the unbinding of pMHC. Subsequently, they return to the basal state (T) with a rate of $\lambda$.

$$\frac{dL}{dt} = -\kappa LR + v\sum_{i=0}^{N} C_i - \kappa LR^* \tag{49}$$

$$\frac{dR}{dt} = -\kappa LR + v\sum_{i=0}^{N-1} C_i + \Omega R^* \tag{50}$$

$$\frac{dR^*}{dt} = vC_N - \kappa LR^* - \Omega R^* \tag{51}$$

$$\frac{dC_0}{dt} = \kappa LR - (v+\phi)C_0 \tag{52}$$

$$\frac{dC_i}{dt} = \phi C_{i-1} - (\phi+v)C_i; \quad 1 \le i \le N-1 \tag{53}$$

$$\frac{dC_N}{dt} = \phi C_{N-1} - vC_N + \kappa LR^* \tag{54}$$

The conservation equations are:

$$L_T = L + C_T \quad R_T = R + C_T + R^*$$

$$\text{T cell activation} = C_N + R^*$$

From equation (51) at the steady state

$$R^* = \frac{vC_N}{\kappa L + \Omega} \tag{55}$$

Also substituting (55) into (50) at steady state we get

$$-(v+\phi)C_0 + v\sum_{i=0}^{N-1} C_i + \Omega R^* = 0$$

$$-(v+\phi)C_0 + v\sum_{i=0}^{N-1} a^i C_0 + \Omega\frac{vC_N}{\kappa L + \Omega} = 0$$

$$-(v+\phi)C_0 + v\frac{1-a^n}{1-a}C_0 + \Omega\frac{vC_N}{\kappa L + \Omega} = 0$$

upon rearrangement and solving we get

$$C_0 = \frac{\Omega(1-a)}{a^n(\kappa L + \Omega)}C_N$$

Also,

$$\begin{aligned} C_T &= \sum_{i=0}^{N} C_i \\ &= \sum_{i=0}^{N-1} C_i + C_N \\ &= \sum_{i=0}^{N-1} a^i C_0 + C_N \\ &= \frac{1-a^n}{1-a}C_0 + C_N \\ &= \left(\frac{1-a^n}{1-a}\right)\frac{\Omega(1-a)}{a^n(\kappa L+\Omega)}C_N + C_N \end{aligned}$$

Solving this we get:

$$C_N = \frac{\kappa L + \Omega}{\Omega + a^n \kappa L}a^n C_T \tag{56}$$

Hence from equation (55) and (56), T cell activation is given by

$$\begin{aligned} &\text{T cell activation} \\ &\qquad = C_N + R^* \\ &\qquad = \frac{\kappa L + \Omega}{\Omega + a^n\kappa L}a^n C_T + \frac{vC_N}{\kappa L + \Omega} \\ &\qquad = \left(\frac{\kappa L + v + \Omega}{\Omega + a^n \kappa L}\right)a^n C_T \end{aligned}$$

**Calculating the $E_{\max}$**

Similar to the case of kinetic proofreading model, it can be shown that when $L_T \to \infty$ $C_T \to R_T$ where $C_T$ is given by:

$$C_T = \frac{L_T + R_T + K_D - \sqrt{(L_T + R_T + K_D)^2 - 4L_T R_T}}{2}$$

$$\begin{aligned}
E_{\max} &= \lim_{L_T \to \infty} \left( \frac{\kappa L + v + \Omega}{\Omega + a^n \kappa L} \right) a^n C_T \\
&= \lim_{L_T \to \infty} \left( \frac{\kappa (L_T - C_T) + v + \Omega}{\Omega + a^n \kappa (L_T - C_T)} \right) a^n C_T \\
&= a^n R_T \lim_{L_T \to \infty} \left( \frac{\kappa (1 - C_T/L_T) + v/L_T + \Omega/L_T}{\Omega/L_T + a^n \kappa (1 - C_T/L_T)} \right) \\
&= R_T
\end{aligned}$$

Hence $E_{\max} = R_T$

**Calculating the $EC_{50}$**

At half the maximal response
T cell activation

$$\frac{E_{\max}}{2} = \frac{R_T}{2}$$

which implies

$$\frac{R_T}{2} = \left( \frac{\kappa (L_T - C_T) + v + \Omega}{\Omega + a^n \kappa (L_T - C_T)} \right) a^n C_T$$

Rearranging and solving for $L_T$ we get:

$$L_T = \left( \frac{a^n C_T (v + \Omega - \kappa R_T/2) - \Omega R_T/2}{a^n \kappa (R_T/2 - C_T)} \right)$$

## 2.5 Kinetic proofreading with negative feedback

Kinetic Proofreading with Negative Feedback extends the KPR scheme by introducing the notion that the rates of complex formation in the activation chain can be modulated at intermediate stages and/or within the final signaling state $C_N$. This modulation is achieved through a single negative feedback mechanism mediated by Src homology 2 domain phosphatase-1 (SHP-1).

$$\frac{dL}{dt} = -\kappa L R + v \sum_{i=0}^{N} C_i \tag{57}$$

$$\frac{dR}{dt} = -\kappa L R + v \sum_{i=0}^{N} C_i \tag{58}$$

$$\frac{dC_0}{dt} = \kappa L R + (b + \gamma S) C_1 - (\phi + v) C_0 \tag{59}$$

$$\frac{dC_i}{dt} = \phi C_{i-1} + (b + \gamma S) C_{i+1} - (\phi + v + b + \gamma S) C_i; \quad 1 \le i \le N-1 \tag{60}$$

$$\frac{dC_N}{dt} = \phi C_{N-1} - (b + \gamma S + v) C_N \tag{61}$$

$$\frac{dS}{dt} = \alpha C_1 (S_T - S) - \beta S \tag{62}$$

The conservation equations are:

$$L_T = L + C_T \quad R_T = R + C_T$$

Here $C_T = \sum_{i=0}^{N+1} C_i$, and T cell activation is given by $C_N$.

$$\text{T cell activation} = C_N$$

Now $C_N$ can be expressed in form of $C_T$ as in eq (4.17) in [1],

$$C_N = \frac{1 - r_-/r_+}{1 - (r_-/r_+)^{N+1}} r_-^N C_T \tag{63}$$

where

$$r_\pm = \frac{\phi + b + \gamma S + v \pm \sqrt{(\phi + b + \gamma S + v)^2 - 4\phi(b + \gamma S)}}{2(b + \gamma S)}$$

Therefore, the cell activation is given by

$$\text{T cell activation} = \frac{1 - r_-/r_+}{1 - (r_-/r_+)^{N+1}} r_-^N C_T \tag{64}$$

$$\tag{65}$$

**Calculation for $E_{\max}$**
Similar to the case of kinetic proofreading model, it can be shown that when $L_T \to \infty$ $C_T \to R_T$ where $C_T$ is given by:

$$C_T = \frac{L_T + R_T + K_D - \sqrt{(L_T + R_T + K_D)^2 - 4L_T R_T}}{2}$$

$$\begin{aligned} E_{\max} &= \lim_{L_T \to \infty} \left( \frac{1 - r_-/r_+}{1 - (r_-/r_+)^{N+1}} \right) r_-^N C_T \\ &= \left( \frac{1 - r_-/r_+}{1 - (r_-/r_+)^{N+1}} \right) r_-^N R_T \end{aligned}$$

**Calculation for $EC_{50}$**
At half the maximal response
T cell activation

$$\frac{E_{\max}}{2} = \left( \frac{1 - r_-/r_+}{1 - (r_-/r_+)^{N+1}} \right) \frac{r_-^N R_T}{2}$$

$$\left( \frac{1 - r_-/r_+}{1 - (r_-/r_+)^{N+1}} \right) r_-^N C_T = \left( \frac{1 - r_-/r_+}{1 - (r_-/r_+)^{N+1}} \right) \frac{r_-^N R_T}{2}$$

which implies

$$C_T = \frac{R_T}{2}$$

Since, $L_T = L + C_T \quad R_T = L + C_T$, using this equation in equation (57)

$$\kappa(L_T - C_T)(R_T - C_T) - v\sum_{i=0}^{N} C_i = 0$$
$$(L_T - C_T)(R_T - C_T) = K_D C_T$$
$$L_T = K_D + R_T/2$$

## 2.6 Kinetic proofreading with stabilizing activation chain

In this model a pMHC ligand (L) binds to a TCR receptor (R) to form a pMHC-TCR complex $C_0$, which undergoes series of chemical modifications to reach signalling competent state $C_N$.

This stabilization/destabilization of the $C_i$ complexes and variation in the time taken for activation, are articulated by variations in the values of corresponding rate constants $v(i); (i = 0, 1, ..., N)$ and $\phi(i); (i = 0, 1, ..., N-1)$ as the proofreading progresses.

$$\frac{dL}{dt} = -\kappa LR - \sum_{i=0}^{N} v(i)C_i \tag{66}$$

$$\frac{dR}{dt} = -\kappa LR - \sum_{i=0}^{N} v(i)C_i \tag{67}$$

$$\frac{dC_0}{dt} = \kappa LR - (\phi(0) + v(0))C_0 \tag{68}$$

$$\frac{dC_i}{dt} = \phi(i-1)C_{i-1} - (\phi(i) + v(i))C_i; \quad 1 \leq i \leq N-1 \tag{69}$$

$$\frac{dC_N}{dt} = \phi(N-1)C_{N-1} - v(N)C_N \tag{70}$$

$$v(i) = \frac{(1+i)}{(1+ri)}v; r > 1 \qquad \phi(i) = \phi r^i; r > 1$$

where $v(0) = 1.5$ and $\phi(0) = 1.3$. In this model for simplicity $v(0)$ is denoted as $v(= 1/\tau)$ which is dissociation time, and $\phi(0)$ denotes the propagation rate for first step $C_0 \to C_1$. Now for next steps these rates are given by

$$v(i) = \frac{(1+i)}{(1+ri)}v; r > 1 \qquad \phi(i) = \phi r^i; r > 1$$

The conservation equations are:

$$L_T = L + C_T \quad R_T = R + C_T \quad C_T = \sum_{i=0}^{N} C_i$$

Here we have from [2]

$$C_0 = \frac{C_T}{\mu}; \quad C_i = \gamma C_0, 1 \leq i \leq N-1; \quad C_N = \delta C_T$$

where

$$\mu = 1 + \frac{\phi(N-1)}{v(N)}\gamma(N-1) + \sum_{i=0}^{N-1} \gamma_i$$

$$\gamma_i = \alpha_1 \times ... \times \alpha_i = \prod_{j=1}^{i} \alpha_i; \qquad \alpha_i = \frac{\phi(i-1)}{\phi(i) + v(i)}$$

$$\delta = \frac{1}{\mu}\frac{\phi(N-1)}{v(N)}\gamma(N-1)$$

and where $C_T$ is the number of bound receptors or ligands $C_T = \sum_{i=0}^{N} C_i$ which is given by

$$C_T = \frac{2L_T R_T}{L_T + R_T + \epsilon + \sqrt{(L_T + R_T + \epsilon)^2 - 4L_T R_T}}; \qquad \epsilon = \frac{1}{\mu}\frac{v(0) + \phi(0)}{\kappa}$$

$$\text{T cell activation} = C_N$$
$$= \delta C_T$$

**Calculating the $E_{\max}$**
Similar to the case of kinetic proofreading model, it can be shown that when $L_T \to \infty$ $C_T \to R_T$
Hence

$$E_{\max} = \delta R_T$$

**Calculating the $EC_{50}$**
At half the maximal response
T cell activation

$$\frac{E_{\max}}{2} = \delta R_T/2$$

which implies

$$\delta C_T = \delta R_T/2 \quad C_T = R_T/2$$

Since

$$C_T = \frac{2L_T R_T}{L_T + R_T + \epsilon + \sqrt{(L_T + R_T + \epsilon)^2 - 4L_T R_T}}; \qquad \epsilon = \frac{1}{\mu}\frac{v(0) + \phi(0)}{\kappa}$$

Substituting $C_T = R_T/2$ in above equation and solving for $L_T$ we get

$$L_T = 2R_T + 4\epsilon$$

Hence potency

$$Potency = 2R_T + 4\epsilon$$

## 2.7 Kinetic proofreading with incoherent feed forward loop

Although the actual model [3] considered the KPR with limited signalling combined with incoherent feed forward loop. We first considered the KPR with incoherent feed forward loop only for the plotting.

$$\frac{dL}{dt} = -\kappa LR - v\sum_{i=0}^{N} C_i \tag{71}$$

$$\frac{dR}{dt} = -\kappa LR - v\sum_{i=0}^{N} C_i \tag{72}$$

$$\frac{dC_0}{dt} = \kappa LR - (\phi + v)C_0 \tag{73}$$

$$\frac{dC_i}{dt} = \phi C_{i-1} - (\phi + v)C_i; \quad 1 \le i \le N-1 \tag{74}$$

$$\frac{dC_N}{dt} = \phi C_{N-1} - vC_N \tag{75}$$

$$\frac{dY}{dt} = a(m-Y) - bY + \sigma C_N(m-Y) \tag{76}$$

$$\frac{dX}{dt} = c(l-X) - dX + \delta Y(l-X) - \mu C_N X \tag{77}$$

where $C_T = \sum_{i=0}^{N} C_i$, and T cell activation is given by X.

$$C_N = \alpha^n C_T$$

$$Y = \frac{(1+\sigma C_N/a)m}{1+b/a+\sigma C_N/a} \quad X = \frac{(1+\delta Y/c)l}{1+d/c+\delta Y/c+\mu C_N/d}$$

**Calculating the $E_{\max}$**

Similar to the case of kinetic proofreading model, it can be shown that when $L_T \to \infty$ $C_T \to R_T$

Hence

$$\begin{aligned} E_{\max} &= \lim_{L_T\to\infty}\left(\frac{(1+\delta Y/c)l}{1+d/c+\delta Y/c+\mu_\alpha^n C_T/c}\right) \\ &= \left(\frac{(1+\delta Y/c)l}{1+d/c+\delta Y/c+\mu\alpha^n R_T/c}\right) \end{aligned}$$

**Calculating the $EC_{50}$**

At half the maximal response

T cell activation

$$\frac{E_{\max}}{2} = \left(\frac{(1+\delta Y/c)l}{2(1+d/c+\delta Y/c+\mu\alpha^n R_T/c)}\right)$$

which implies

$$\left(\frac{(1+\delta Y/c)l}{1+d/c+\delta Y/c+\mu\alpha^n C_T/c}\right) = \left(\frac{(1+\delta Y/c)l}{2(1+d/c+\delta Y/c+\mu\alpha^n R_T/c)}\right)$$

$$2(1+d/c+\delta Y/c+\mu\alpha^n R_T/c) = 1+d/c+\delta Y/c+\mu\alpha^n C_T$$

Rearranging and solving for $C_T$

$$C_T = 2R_T + \left(\frac{1+d/c+\delta Y/c}{\mu\alpha^n}\right)$$

Since, $L_T = L + C_T \quad R_T = R + C_T$ Using this equation in (71)

$$\kappa(L_T - C_T)(R_T - C_T) - v\sum_{i=0}^{N+1} C_i = 0$$

$$(L_T - C_T)(R_T - C_T) = K_D C_T$$

Using value of $C_T$ in above equation and solving. we get:

$$L_T = \frac{2R_T^2 + 3R_T U - U^2 - 2K_D R_T - UK_D}{R_T + U}$$

where

$$U = \left( \frac{1 + d/c + \delta Y/c}{\mu \alpha^n} \right)$$

## 2.8 Kinetic proofreading with limited signaling and incoherent feed forward loop

This model is an extension of KPR with incoherent feed forward loop, it has been assumed that signalling is limited and after after reaching the signalling competent state the bound TCR transits to non-signalling transit state.

$$\frac{dL}{dt} = -\kappa LR - v \sum_{i=0}^{N+1} C_i \tag{78}$$

$$\frac{dR}{dt} = -\kappa LR - v \sum_{i=0}^{N+1} C_i \tag{79}$$

$$\frac{dC_0}{dt} = \kappa LR - (\phi + v)C_0 \tag{80}$$

$$\frac{dC_i}{dt} = \phi C_{i-1} - (\phi + v)C_i; \quad 1 \leq i \leq N - 1 \tag{81}$$

$$\frac{dC_N}{dt} = \phi C_{N-1} - (v + \xi)C_N \tag{82}$$

$$\frac{dC_{N+1}}{dt} = \xi C_N - vC_{N+1} \tag{83}$$

$$\frac{dY}{dt} = a(m - Y) - bY + \sigma C_N(m - Y) \tag{84}$$

$$\frac{dX}{dt} = c(l - X) - dX + \delta Y(l - X) - \mu C_N X \tag{85}$$

where $C_T = \sum_{i=0}^{N} C_i$, and T cell activation is given by X.
Here

$$C_N = \left( \frac{v}{v + \xi} \right) \alpha^n C_T$$

$$Y = \frac{(1 + \sigma C_N/c)m}{1 + d/c + \sigma C_N/c} \quad X = \frac{(1 + \delta Y/c)l}{1 + d/c + \delta Y/c + \mu C_N/c}$$

**Calculating the $E_{\max}$**
Similar to the case of kinetic proofreading model, it can be shown that when $L_T \to \infty$ $C_T \to R_T$
Hence

$$E_{\max} = \lim_{L_T \to \infty} \left( \frac{(1 + \delta Y/c)l}{1 + d/c + \delta Y/c + v\mu\alpha^n C_T/(v + \xi)c} \right)$$
$$= \left( \frac{(1 + \delta Y/c)l}{1 + d/c + \delta Y/c + v\mu\alpha^n R_T/(v + \xi)c} \right)$$

**Calculating the $EC_{50}$**
At half the maximal response
T cell activation

$$\frac{E_{\max}}{2} = \left( \frac{(1 + \delta Y/c)l}{2(1 + d/c + \delta Y/c + v\mu\alpha^n R_T/(v + \xi)c)} \right)$$

which implies

$$\frac{(1+\delta Y/c)l}{1+d/c+\delta Y/c+v\mu\alpha^n C_T/(v+\xi)c} = \left(\frac{(1+\delta Y/c)l}{2(1+d/c+\delta Y/c+v\mu\alpha^n R_T/(v+\xi)c)}\right)$$

$$2(1+d/c+\delta Y/c+v\mu\alpha^n R_T/(v+\eta)c) = 1+d/c+\delta Y/c+v\mu\alpha^n C_T/(v+\xi)c$$

Rearranging and solving for $C_T$

$$C_T = 2R_T + \left(\frac{(c+d+\delta Y)(v+\xi)}{\mu v\alpha^n}\right)$$

Since, $L_T = L + C_T \quad R_T = R + C_T$, using this equation in (78)

$$\kappa(L_T - C_T)(R_T - C_T) - v\sum_{i=0}^{N} C_i = 0$$

$$(L_T - C_T)(R_T - C_T) = K_D C_T$$

Using value of $C_T$ in above equation and solving. we get:

$$L_T = \frac{2R_T^2 + 3R_T W - W^2 - 2K_D R_T - WK_D}{R_T + W}$$

where

$$W = \left(\frac{(c+d+\delta Y)(v+\xi)}{\mu v\alpha^n}\right)$$

## 2.9 Kinetic proofreading with Limited and Sustained Signaling

It as a combination of two models KPR with limited signaling and KPR with sustained signaling.

$$\frac{dL}{dt} = -\kappa LR - \kappa LR^* - v\sum_{i=0}^{N+1} C_i \tag{86}$$

$$\frac{dR}{dt} = -\kappa LR + v\sum_{i=0}^{N-1} C_i + vC_{N+1} + \Omega R^* \tag{87}$$

$$\frac{dC_0}{dt} = \kappa LR - (\phi + v)C_0 \tag{88}$$

$$\frac{dC_i}{dt} = \phi C_{i-1} - (\phi + v)C_i; \quad 1 \le i \le N-1 \tag{89}$$

$$\frac{dC_N}{dt} = \phi C_{N-1} - (v+\xi)C_N + \kappa LR^* \tag{90}$$

$$\frac{dC_{N+1}}{dt} = \xi C_N - vC_{N+1} \tag{91}$$

$$\frac{dR^*}{dt} = vC_N - \kappa LR^* - \Omega R^* \tag{92}$$

The conservation equations are:

$$L_T = L + C_T \quad R_T = R + C_T + R^*$$

where $C_T = \sum_{i=0}^{N+1} C_i$

$$\text{T cell activation} = C_N + R^*$$

From (92) at the steady state

$$R^* = \frac{vC_N}{\kappa L + \Omega} \tag{93}$$

Now, using it and (88) at a steady state; for (87) at a steady state we have

$$-(\phi + v)C_0 + v\sum_{i=0}^{N-1} C_i + vC_{N+1} + \Omega\frac{vC_N}{\kappa L + \Omega} = 0$$

$$-(\phi + v)C_0 + vC_T - vC_N + \Omega\frac{vC_N}{\kappa L + \Omega} = 0$$

$$C_0 = \frac{v}{(\phi + v)}C_T - \frac{v}{(\phi + v)}C_N + \frac{v}{(\phi + v)}\frac{\Omega C_N}{\kappa L + \Omega}$$

$$C_0 = (1-a)C_T - (1-a)C_N + (1-a)\frac{\Omega C_N}{\kappa L + \Omega}; \qquad \text{where} \quad a = \frac{\phi}{(\phi + v)}$$

As $C_T$ is given by:

$$\begin{aligned}
C_T &= \sum_{i=0}^{N+1} C_i \\
&= \sum_{i=0}^{N-1} C_i + C_N + C_{N+1} \\
&= \sum_{i=0}^{N-1} a^i C_0 + C_N + \frac{\xi}{v}C_N \\
&= \frac{1-a^n}{1-a}C_0 + C_N + \frac{\xi}{v}C_N \\
&= \left(\frac{1-a^n}{1-a}\right)\left((1-a)C_T - (1-a)C_N + (1-a)\frac{\Omega C_N}{\kappa L + \Omega}\right) + C_N + \frac{\xi}{v}C_N \\
&= \frac{1}{a^n}\frac{(a^n\kappa L + \Omega v + \xi\kappa L + \xi\Omega)}{v(\kappa L + \Omega)}C_N
\end{aligned}$$

Which implies $C_N$ is given by:

$$C_N = \frac{v(\kappa L + \Omega)}{(a^n\kappa L + \Omega v + \xi\kappa L + \xi\Omega)}a^n C_T \tag{94}$$

$$\begin{aligned}
&\text{T cell activation} \\
&\qquad = C_N + R^* \\
&\qquad = C_N + \frac{vC_N}{\kappa L + V} \\
&\qquad = \frac{\kappa L + \Omega + v}{\kappa L + \Omega}C_N \\
&\qquad = \frac{v(\kappa L + \Omega + v)}{(a^n\kappa L + \Omega v + \xi\kappa L + \xi\Omega)}a^n C_T
\end{aligned}$$

**Calculating the** $E_{\max}$
Similar to the case of kinetic proofreading model, it can be shown that when $L_T \to \infty$ $C_T \to R_T$ where $C_T$ is given by:

$$C_T = \frac{L_T + R_T + K_D - \sqrt{(L_T + R_T + K_D)^2 - 4L_T R_T}}{2}$$

$$\begin{aligned}
E_{\max} &= \lim_{L_T \to \infty} \left( \frac{v(\kappa L + \Omega + v)}{(a^n \kappa L + \Omega v + \xi \kappa L + \xi \Omega)} \right) a^n C_T \\
&= \lim_{L_T \to \infty} \left( \frac{v(\kappa (L_T - C_T) + \Omega + v)}{(a^n \kappa (L_T - C_T) + \Omega v + \xi \kappa (L_T - C_T) + \xi \Omega)} \right) a^n C_T \\
&= a^n R_T \lim_{L_T \to \infty} \left( \frac{v(\kappa (1 - C_T/L_T) + \Omega/L_T + v/L_T)}{(a^n \kappa (1 - C_T/L_T) + \Omega v/L_T + \xi \kappa (1 - C_T/L_T) + \xi \Omega/L_T)} \right) \\
&= \frac{v}{a^n + \xi} a^n R_T
\end{aligned}$$

**Calculating the** $EC_{50}$ At half the maximal response
T cell activation

$$\frac{E_{\max}}{2} = \frac{\frac{v}{a^n+\xi} a^n R_T}{2}$$

which implies

$$\frac{\frac{v}{a^n+\xi} a^n R_T}{2} = \left( \frac{v(\kappa (L_T - C_T) + \Omega + v)}{(a^n \kappa (L_T - C_T) + \Omega v + \xi \kappa (L_T - C_T) + \xi \Omega)} \right) a^n C_T$$

Rearranging and solving for $L_T$ we get:

$$L_T = \frac{R_T (a^n \kappa C_T - \Omega v + \xi \kappa C_T - \xi \Omega) + 2(a^n + \xi)(\Omega + v - \kappa C_T)}{R_T a^n \kappa + \xi \kappa R_T - 2(a^n + \xi) \kappa C_T}$$

## References


[1] A. D. Rendall and E. D. Sontag. "Multiple steady states and the form of response functions to antigen in a model for the initiation of T-cell activation". *Royal Society Open Science* 4.11 (2017), p. 170821. DOI: https://doi.org/10.1098/rsos.170821.

[2] J. Gálvez, J. J. Galvez, and P. Garcia-Penarrubia. "TCR/pMHC interaction: phenotypic model for an unsolved enigma". *Frontiers in Immunology* 7 (2016), p. 467. DOI: https://doi.org/10.3389/fimmu.2016.00467.

[3] M. Lever et al. "Architecture of a minimal signaling pathway explains the T-cell response to a 1 million-fold variation in antigen affinity and dose". *Proceedings of the National Academy of Sciences* 113.43 (2016), E6630–E6638. DOI: https://doi.org/10.1073/pnas.1608820113.
